# Reporting of activated partial thromboplastin time (aPTT): Could we achieve better comparability of the results?

**DOI:** 10.11613/BM.2021.020708

**Published:** 2021-06-15

**Authors:** Ana Bronić, Sandra Margetić, Desiree Coen Herak, Marija Milić, Branka Krešić, Vanja Radišić Biljak, Jasna Leniček Krleža

**Affiliations:** 1 Clinical Institute of Chemistry, Sestre milosrdnice University Hospital Center, Zagreb, Croatia; 2 Working Group for Laboratory Coagulation of the Croatian Society of Medical Biochemistry and Laboratory Medicine; 3 Department of Laboratory Diagnostics, University Hospital Centre Zagreb, Zagreb, Croatia; 4 Department of Clinical Laboratory Diagnostics, University Hospital Centre Osijek, Osijek, Croatia; 5 Faculty of Medicine, Josip Juraj Strossmayer University of Osijek, Osijek, Croatia; 6 Department of Medical Laboratory Diagnostics, University Hospital Centre Split, Split, Croatia; 7 Department of Medical Laboratory Diagnostics, University Hospital “Sveti Duh”, Zagreb, Croatia; 8 Department of Laboratory Diagnostics, Children’s Hospital Zagreb, Zagreb, Croatia; 9 Croatian Centre for Quality Assessment in Laboratory Medicine (CROQALM)

**Keywords:** activated partial thromboplastin time, external quality assessment, harmonization

## Abstract

**Introduction:**

Activated partial thromboplastin time (aPTT) is determined and reported as clotting time in seconds aPTT(s), but it is presumed that reporting results as patient-to-normal clotting time ratio, aPTT(r), could minimize within-laboratory variability. The aim of study was to investigate differences in reporting aPTT results that can affect comparability of the results among Croatian laboratories and suggest further steps for its harmonization.

**Materials and methods:**

The questionnaire on aPTT reporting practice was distributed to 83 laboratories through Survey Monkey application in March 2019 as the part of the first regular round of Croatian Centre for Quality Assessment in Laboratory Medicine proficiency testing.

**Results:**

The survey response rate was 0.49. Majority of laboratories report aPTT results as both, seconds and ratio. Participants reported use of 23 different aPTT(s) reference intervals along with 17 different combinations of reagent/coagulometer and 25 aPTT(s) denominators of different origin for aPTT(r) calculation. Despite the same aPTT(s) results, the use of different denominators caused a dispersion of aPTT(r) results that can lead to exceeding external quality assessment performance criteria of 7%, particularly when results were compared for the same reagent group only. By applying aPTT(s) reference interval mean as denominator for calculation of aPTT(r) reference interval better concordance to harmonized one was obtained (17 *vs*. 27; χ^2^ = 3.972; P = 0.046).

**Conclusion:**

In order to improve comparability of the results, laboratories are advised to use mean of aPTT(s) reference interval as denominator for aPTT(r) calculation. Type of coagulometer need to be considered when evaluating aPTT proficiency test results and its currently acceptable limit of performance evaluated accordingly.

## Introduction

Activated partial thromboplastin time (aPTT) is a coagulation screening assay that estimates the activity of the intrinsic and common coagulation pathway. The assay is used to screen for coagulation factor deficiency, monitor unfractionated heparin (UFH) therapy and detect the presence of lupus anticoagulant (LA) (*1*). Responsiveness of commercially available aPTT reagents on these pathophysiological conditions is different as reagents contain different types and concentrations of activators and phospholipids (*2*). Besides, results are highly dependent on the combination of reagent and coagulometer in use, making results comparison difficult. Therefore, it is not surprising that there is still no global assay standardization.

Activated partial thromboplastin time is determined and reported as clotting time in seconds (aPTT(s)). Reporting of the results as aPTT ratio (aPTT(r)) of patient-to-normal clotting time is often used in terms of improving comparability of the results between different laboratories, especially for UFH dose-adjustment or laboratory diagnosis of LA (*3*). Although it is presumed that reporting the aPTT(r) has an advantage in reducing within-laboratory variability, manufacturers usually do not provide complete information for calculation of aPTT(r) (*4*). What is considered as “normal” clotting time in patient-to-normal clotting time calculation usually is not defined (*3*, *4*).

In 2005, Croatian Chamber of Medical Biochemists (CCMB) recommended reporting of aPTT as ratio, with a harmonized reference interval of 0.8-1.2 (*5*). As a sequel to it, in 2006, Commission for External Quality Assessment of the Croatian Society of Biochemists (predecessor of Croatian Centre for Quality Assessment in Laboratory Medicine, CROQALM), instructed laboratories to report aPTT(r) along with aPTT(s), and specified that it should be calculated by dividing the aPTT(s) result with the mean value of reference interval that laboratories use for aPTT(s) (*6*). CROQALM assesses performance of both, aPTT(s) and aPTT(r). Reporting of the results in both ways was also recommended in recently published national guidelines (*7*). According to literature, different external quality assessment (EQA) providers assess performance of aPTT in seconds, ratio or in both ways, applying quite different acceptable limits of performance, ranging from 15 to 25% (*8*, *9*). Acceptable bias of 7% for aPTT result assessment, used by CROQALM from 2017, originates from biological variability database (*10*, *11*). Thus, in addition to lack of standardization of aPTT assay as well as result reporting there is also no consensus on aPTT performance criteria.

The aim of study was to investigate differences in reporting aPTT results that can affect comparability of the results among Croatian laboratories and suggest further steps for its harmonization.

## Materials and methods

### Questionnaire design

The Working Group for Laboratory Coagulation (WGLC) operating within the Committee for scientific and professional development of the Croatian Society of Medical Biochemistry and Laboratory Medicine (CSMBLM), in cooperation with the CROQALM created a questionnaire that was distributed to laboratories performing aPTT assay through Survey Monkey application (SurveyMonkey Inc., Palo Alto, California, USA) in March 2019 as the part of the first regular CROQALM proficiency testing round. Participation in the survey was voluntary.

The questionnaire comprised 9 questions on: reference intervals for aPTT(s) and aPTT(r), detailed data on population used for the determination of reference interval provided by the manufacturer, values used to calculate aPTT(r) and coefficients of variation (CV) on normal and pathological internal commercial control levels at last month (Supplement 1). Statements were created by all authors and reflected personal impression about the most problematic issues related to the comparability of aPTT results. The survey was entitled as Questionnaire of WGLC and it was part of the Module 4 (https://www.surveymonkey.com/r/PXL6QKP). Additional data collected from the CROQALM for each laboratory that responded the survey were the combinations of aPTT reagent and coagulometer in use. Furthermore, reference guides or assay sheets were reviewed in order to complete and compare information on reference intervals for different combinations of reagent and coagulometer in use.

### Statistical analysis

Data for analysis were obtained by counting and calculating frequency of answers by using Microsoft Excel 2010 (Microsoft, Redmond, Washington, USA) program. Results are reported as counts and proportions (N<100). A comparison between groups was performed by Chi square test. The value of P<0.05 was considered statistically significant. Data analyses were performed by using statistical software MedCalc 11.5.1. (MedCalc Software, Ostend, Belgium).

## Results

In the first round of CROQALM proficiency testing in 2019, 83 laboratories reported results for aPTT test. After removing duplicate responds from the final data processing, 41 surveys were considered for the analysis, resulting in the response rate of 0.49. No additional exclusion criteria were applied.

### Reporting of aPTT results

Among all survey participants, 35/41 reported aPTT results both as seconds and ratio, 1/41 reported only aPTT(s), whereas 3/41 reported results only as aPTT(r).

#### Reference intervals - aPTT(s)

In total, 39 laboratories declared the use of 23 different reference intervals for aPTT(s) along with 17 different combinations of coagulometers and aPTT reagents (Table 1). For reporting aPTT(s), laboratories mainly use reference interval assigned by the manufacturer (N = 22), whereas literature data (N = 12) or own reference intervals (N = 4) are less in use. Only 22/41 participants responded that manufacturers provide information on population used for setting up aPTT(s) reference interval. When laboratories were asked to specify exact details, 5 laboratories stated that only number of subjects is provided, 14 mean values, 7 median and 10 combinations of all the above mentioned. Results related to reviewing reference guides and assay sheets revealed that in addition to the number of participants used to determine aPTT(s) reference interval, both, population mean and median aPTT(s) value is given by manufacturer for reagent/coagulometer combination used by 34 survey participants.

**Table 1:** Combination of aPTT reagents, different coagulometers and reference intervals in use among survey participants

				**Survey results: data reported by laboratories**				**Calculated data**	
**Manufacturer**	**Reagent**	**Coagulometer in use**	**N**	**Reference interval** **in use [aPTT (s)]**	**Value of aPTT(s) used** **for aPTT(r) calculation**	**Value of aPTT(s) used for calculation represent**	**Reference interval in use [aPTT (r)]**	**Reference interval [aPTT (r)] calculated according data reported by laboratories**	**Reference interval [aPTT (r)] calculated with mean value of aPTT(s) RI as denominator**
**Siemens**	**Actin FS**	**BCS XP**	**10**	22.0-33.0	28.0	RI-mean	0.8-1.2	0.8-1.2	0.8-1.2
				23.0-32.0	26.8	M-Median	0.8-1.2	0.9-1.2	0.8-1.2
				24.0-33.0	28.5	RI-mean	0.8-1.2	0.8-1.2	0.8-1.2
				23.0-32.0	26.8	M-Median	0.8-1.2	0.9-1.2	0.8-1.2
				23.0-32.0	27.5	Mean	0.8-1.2	0.8-1.2	0.8-1.2
				23.0-32.0	27.5	RI-mean	0.8-1.2	0.8-1.2	0.8-1.2
				23.0-32.0	27.5	RI-mean	0.8-1.2	0.8-1.2	0.8-1.2
				23.0-32.0	27.5	RI-mean	0.8-1.2	0.8-1.2	0.8-1.2
				23.0-31.9	27.45	RI-mean	0.8-1.2	0.8-1.2	0.8-1.2
				21.8-32.6	26.8	M-Median	0.8-1.2	0.8-1.2	0.8-1.2
		**BFT II**	**5**	lab does not report seconds	24.5	M-mean	0.8-1.2	0.9-1.2	0.9-1.1
				23.0-31.9	27.45	RI-mean	0.8-1.2	0.8-1.2	0.8-1.2
				22.4-29.3	26.2	M-mean	0.8-1.2	0.9-1.1	0.9-1.1
				24.3-35.0	29.6	RI-mean	0.8-1.2	0.8-1.2	0.8-1.2
				22.4-29.3	26.2	M-mean	0.8-1.2	0.9-1.1	0.9-1.1
		**CA-500**	**3**	22.0-28.0	24.7	M-Median	0.8-1.2	0.9-1.1	0.9-1.1
				22.0-37.0	25.5	M-Median	0.8-1.2	0.9-1.5	0.8-1.2
				22.0-28.0	25.0	M-Median	0.8-1.2	0.9-1.1	0.9-1.1
		**CA-560**	**6**	21.8-28.0	24.6	M-Median	0.8-1.2	0.9-1.1	0.9-1.1
				21.8-28.0	25.6	M-mean	0.8-1.2	0.9-1.1	0.9-1.1
				21.8-28.0	24.0	M-mean	0.8-1.0	0.9-1.2	0.9-1.1
				21.8 - 28.0	24.8	M-mean	0.8-1.2	0.9-1.1	0.9-1.1
				21.8-28.0	24.8	M-mean	0.8-1.2	0.9-1.1	0.9-1.1
				23.0-33.0	28.0	RI-mean	0.8-1.2	0.8-1.2	0.8-1.2
		**CA-620**	**1**	lab does not report seconds	24.6	M-Median	0.8-1.2	0.9-1.2	0.9-1.1
		**CA-660**	**2**	22.0-28.0	25.0	RI-mean	0.8-1.2	0.9-1.1	0.9-1.1
				24.0-33.0	28.5	RI-mean	0.8-1.2	0.8-1.2	0.8-1.2
		**CS 2000i**	**2**	21.6-28.7	25.2	RI-mean	0.9-1.1	0.9-1.1	0.9-1.1
				22.0-33.0	24.2	M-mean	0.8-1.2	0.9-1.4	0.8-1.2
		**CS-2500**	**1**	25-60 (< 5 days) 32-50 (6 d-3m) 28-43 (3-6 m) 23-36 (> 6 m)	26.6	RI-mean		0.9-1.4	0.9-1.4
**Siemens**	**Pathromtin SL**	**BFT II**	**1**	22.1-28.1	34.1	M-Median	0.8-1.2	0.8-1.2	0.8-1.2
		**BCS XP**	**1**	25.0-40.0	32.5	RI-mean	0.8-1.2	0.8-1.2	0.8-1.2
		**CA-660**	**1**	21.0-33.0	lab do not report aPTT(r)	NA	NA	0.8-1.2	0.8-1.2
**Diagnostica Stago**	**STA Cephascreen**	**STA Satellite**	**1**	22.0-36.0	32.4	Manufacturer value	0.8-1.2	0.7-1.0	0.8-1.2
		**STA Compact Max**	**1**	24.0-34.0	32.7	M-mean	0.8-1.2	0.7-1.1	0.8-1.2
	**APTT–automate**	**STA Satellite**	**1**	25.0-38.0	29.2	M-mean	0.8-1.2	0.9-1.3	0.8-1.2
Sclavo Diagnostics	**Sclavo aPTT S**	**CA-500**	**1**	23.0-35.0	29.0	RI-mean	0.8-1.2	0.8-1.2	0.8-1.2
Spinreact	**APTT Spinreact**	**Bio Bas 1**	**1**	23.0-35.0	28.0	RI-mean	0.8-1.2	0.8-1.3	0.8-1.2
Human	**HemoStat** **APTT-EL**	**Humaclot Duo**	**1**	lab does not report seconds	34.7	M-Median	0.8-1.2	0.9-1.3	0.8-1.2
**Other**	**Not stated**	**Not stated**	**2**	21.8-32.6	24.7	RI-mean	0.9-1.1	0.9-1.2	0.8-1.2
		**Not stated**		26.0-36.0	29.8	Not given	Not given	0.8-1.2	0.8-1.2
N - number of laboratories. aPTT(s) - activated partial thromboplastin time reported in seconds. aPTT(r) - activated partial thromboplastin time reported as ratio. RI - reference interval. M-mean - manufacturer mean. M-Median - manufacturer median. NA - not applicable. d -days. m - months. M - male.									

#### Reference intervals - aPTT(r)

Three different reference intervals for reporting aPTT(r) results are in use among survey participants (Table 1). Most of them reported use of harmonized reference interval (N = 30) given by CCMB, but for the calculation of aPTT(r) even 25 different aPTT(s) values from different sources were listed. Mean or median value of the population for which reference interval was established is used by 11 and 10 participants, respectively, whereas the mean of the reference interval in use by 17 participants.

Reported aPTT(s) reference interval limits were divided by the denominator that participants use for aPTT(r) calculation in order to check if aPTT(r) reference interval limits used by laboratories are aligned and compatible with the harmonized interval. Compared to harmonized interval a wider range of aPTT(r) limits (0.7 to 1.4) was obtained.

Results revealed that 17 aPTT(r) reference intervals are completely in concordance to harmonized reference interval (Table 1). However, by using the aPTT(s) reference interval mean as denominator, a significant increase in aPTT(r) concordant reference intervals was obtained (N = 27; χ^2^ = 3.972; P = 0.046) (Table 1).

An example of aberration obtained by applying different denominators (mean, median and mean of reference interval) is given in Table 2.

**Table 2:** An example of calculating aPTT(r) reference interval by using different denominators obtained from manufacturers reference guides or assay sheets

**A. An example of data on population used to obtain reference interval that is reported in manufacturers reference guide for the particular reagent/coagulometer combination:**					
	**N**	**Mean**	**Median**		**5th - 95th percentile**
**aPTT(s)**	111	27.2	26.8		23.0-31.9
**B. Calculated mean of aPTT(s) reference interval suggested to be used by manufacturer:**					
27.45 (rounded to) 27.5					
**C. Examples of calculation of aPTT(r) reference interval limits by using different denominators that could be obtained from manufacturers reference guides:**					
**Denominator is:**				**Value of aPTT(s) denominator**	**aPTT(r) limits obtained from aPTT(s)**
**Mean**				27.2	0.84-1.17 (0.8-1.2)
**Median**				26.8	0.86-1.19 (0.9-1.2)
**Mean of manufacturer reference interval**				27.5	0.83-1.16 (0.8-1.2)
aPTT(r) - activated partial thromboplastin time reported as ratio. aPTT(s) - activated partial thromboplastin time reported in seconds.					

#### Comparability between laboratories

In the first round of CROQALM proficiency testing in 2019, the provider calculated and reported results for experimental groups with more than five participants, depending on aPTT reagents in use for normal plasma range. Obtained results of CVs in groups that use Siemens Actin FS (N = 62), Siemens Pathromtin SL (N = 5) and Stago STA Cephascreen (N = 7) were 5.4%, 4.2% and 2.2%, respectively. Thus, these laboratories successfully meet criteria of 7% for aPTT(s) and the final conclusion was that 87-100% participants successfully completed the cycle. Results were not assessed according to type of analyser in use.

Among all participants, 33/41 laboratories reported analytical CV for aPTT(s) of internal control samples, obtained for a month prior to the proficiency testing cycle, from 1.2 to 7.0% at the pathological control level, and 36 laboratories reported CVs at normal level from 1.1 to 10.1%. Also, 5 of 41 laboratories did not reported CVs as they stated that for control material they use the aPTT target values are not provided by the manufacturer.

## Discussion

The aim of this survey was to investigate factors that contribute to differences in reporting aPTT results among laboratories in Croatia and to investigate between-laboratory comparability improvement. Survey results revealed that laboratories that use same combinations of reagent/coagulometer for calculation of aPTT(r), often use denominators of different origin. Consequently, aPTT(s) and aPTT(r) reference intervals in use are not always mutually aligned. Use of different denominators for calculating aPTT(r) values complicate comparability of the results and such dispersion of aPTT(r) results may lead to failure to meet the EQA performance criteria despite the same aPTT(s) results.

This is not surprising as reference guides or package inserts mainly report measured mean and/or median value of aPTT(s) in population used to establish provided reference interval, but the mean value of the aPTT(s) reference interval is often slightly different from the population mean and manufacturer instructions for aPTT(r) calculation are mainly vague. Our analysis showed that by applying different denominators for the aPTT(r) calculation, different aPTT(r) reference limits, often exceeding allowable limit of 7% could be generated for the same combination of reagent and coagulometer in use.

Furthermore, CROQALM conducts three proficiency testing cycles each year, distributing to participants two samples at pathological level and one sample at the normal level. Although laboratories mainly successfully met the criteria at normal level (> 95% participants successfully completed the cycles in 2017, 2018 and first cycle in 2019), only 43-67% laboratories successfully met the criteria of 7% at the pathological level (*10*, *11*). Survey participants reported wide range of internal quality control of analytical CVs for aPTT(s) they obtained on their reagent to coagulometer combination in the month prior to the first round of proficiency testing in 2019, some of which exceeded 7%. As aPTT(s) reference interval for the same aPTT reagent differ for different analytical systems, it is not uncommon for this limit to be exceeded when EQA results are compared for the same reagent group only. Reviewing the proficiency testing results for aPTT, obtained by other EQA providers, different approach for result assessment could be found (*8*-*11*). Different providers assess differently reported results (exclusively as seconds or as ratio, or both), and also apply different acceptable limits of the performance (*8*, *9*). Results are assessed according to system in use (reagent/coagulometer) and allowable limit is mainly set up in between 15 and 25% (*8*, *9*). The acceptable limit of 7% used by CROQALM originates from biological variability database and was set up in 2017 to assess performance of the aPTT results reported as both, seconds and ratio (*10*). The provider referred it to different aPTT reagents in use, but without taking into consideration combination of the reagents and coagulometer. In the study of Olson and colleagues, eight different analytical approaches were used by eleven EQA organizations that participated in the study. Authors concluded that there is lack of agreement of pass/fail grading among EQA programs for 218 laboratories. Discordance in the grading was 17.9% and 11% of normal and prolonged aPTT results, respectively (*8*). Thus, international consensus yet needs to be achieved.

To illustrate the impact of coagulometer on aPTT results, an example reflecting similar conditions to EQA was obtained from a clinical laboratory operating at three different locations that uses the same aPTT reagent on two different types of coagulometers is given (Supplement 2). Two times a month, the same patient sample is analysed at each location. Results obtained by using same reagents, but different coagulometers cannot meet the criteria of 7% in comparison to results obtained on the same coagulometers placed on different locations.

This is often problematic not only for aPTT expressed in seconds, but especially for the results expressed as aPTT(r) (Supplement 2). Thus, it could be recommended that EQA results should be assessed according to combination of reagent and type of coagulometer in use.

Another problem stemming from these comparisons is reporting of the aPTT(r) results with different number of decimal places (Supplement 2). Although reporting aPTT(r) at different number of decimal places have no clinical significance and should be viewed only in the light of uniformity, by applying allowable criteria of 7% significant differences in the results could be generated. This could be problematic especially for accredited laboratories and renewing licence for the assay.

Also, when considering CVs, survey revealed that small proportion of laboratories stated not to have target aPTT values for internal control sample in use. Since problems with aPTT performance could be discovered through unacceptable results of the regularly scheduled quality control it is important to educate these laboratories to introduce internal control samples with aPTT target values into everyday practice.

Better comparability of results was obtained by using the mean of the aPTT(s) reference interval as denominator (*5*). Thus, it could be recommended to the laboratories to use the mean of the applied reference interval for the calculation of aPTT(r).

The main limitation of the study certainly was the survey response rate. Obtained analysis indicated certain critical point that might be important for optimisation of reporting aPTT results among Croatian laboratories, but extensive research through longer period with comprehensive statistical analysis should be also performed.

However, more uniformity of the aPTT(r) calculation is needed. At first, adherence to current recommendations on aPTT results reporting should be improved (*6*, *7*). Laboratories are advised to use the same denominator for aPTT(r) calculation, preferably mean of aPTT(s) reference interval. Type of coagulometer need to be considered when evaluating aPTT proficiency test results and its currently acceptable limit of performance evaluated accordingly.
